# AI as the Therapist: Student Insights on the Challenges of Using Generative AI for School Mental Health Frameworks

**DOI:** 10.3390/bs15030287

**Published:** 2025-02-27

**Authors:** Cecilia Ka Yuk Chan

**Affiliations:** Faculty of Education, The University of Hong Kong, Hong Kong SAR, China; ckchan09@hku.hk

**Keywords:** generative AI (GenAI), school mental health, digital therapy, student perceptions, empathy and trust in AI

## Abstract

The integration of generative AI (GenAI) in school-based mental health services presents new opportunities and challenges. This study focuses on the challenges of using GenAI chatbots as therapeutic tools by exploring secondary school students’ perceptions of such applications. The data were collected from students who had both theoretical and practical experience with GenAI. Based on Grodniewicz and Hohol’s framework highlighting the “Problem of a Confused Therapist”, “Problem of a Non-human Therapist”, and “Problem of a Narrowly Intelligent Therapist”, qualitative data from student reflections were examined using thematic analysis. The findings revealed that while students acknowledged AI’s benefits, such as accessibility and non-judgemental feedback, they expressed significant concerns about a lack of empathy, trust, and adaptability. The implications underscore the need for AI chatbot use to be complemented by in-person counselling, emphasising the importance of human oversight in AI-augmented mental health care. This study contributes to a deeper understanding of how advanced AI can be ethically and effectively incorporated into school mental health frameworks, balancing technological potential with essential human interaction.

## 1. Introduction

Artificial intelligence (AI) can now be found in various aspects of life and has been increasingly utilised in health care (World Health Organisation, 2024), particularly since the public release of generative AI (GenAI) (Chan & Colloton, 2024), to enable advanced, emotionally aware interactions (Li et al., 2023; Vistorte et al., 2024). In the face of increasingly stressful educational environments, AI can provide immediate emotional support alongside other measures aimed at improving teaching, learning, and work environments for the academic community. AI-integrated tools could offer real-time responses, improve accessibility, and act as an alternative to traditional practices (Fiske et al., 2019; Gutierrez et al., 2024; Laranjo et al., 2018). Research has shown that there is growing familiarity with GenAI tools and awareness of their capabilities among students (Abbas et al., 2024; Chan, 2024; Chan & Lee, 2023). However, the usability and trustworthiness of AI in such sensitive roles in emotional support remain uncertain, necessitating a closer examination of how users perceive and interact with AI in a therapeutic context. While AI-based mental health support, particularly with GenAI, has been extensively studied in university settings (Crawford et al., 2024; Fulmer et al., 2018), there is comparatively less empirical research focused on its use in secondary schools. Hence, this study aims to explore the challenges of using the GenAI chatbot as a therapeutic tool from the perspective of secondary school students.

### 1.1. Professional Therapists and Psychotherapy in Schools

In school settings, professional therapists provide a variety of therapeutic services like counselling and psychotherapy to support students’ mental health, emotional well-being, and coping skill development. According to the American Psychological Association (2023), psychotherapy is defined as follows:

“communication between patients and therapists that is intended to help people: (i) find relief from emotional distress, as in becoming less anxious, fearful or depressed, (ii) seek solutions to problems in their lives, such as dealing with disappointment, grief, family issues, and job or career dissatisfaction, and (iii) modify ways of thinking and acting that are preventing them from working productively and enjoying personal relationships.”

However, despite the importance of these services, many schools struggle to meet students’ mental health needs due to limited resources, counsellor shortages, and high demand (National Education Association, 2023). Marsh and Mathur (2020) highlight that professionals such as school counsellors, psychologists, and social workers play crucial roles in identifying students in need of support and coordinating services. However, they also note that these professionals may not consistently recognise mental health issues due to their limited presence in classrooms, which can hinder timely intervention. Marraccini et al. (2023) and Kang-Yi et al. (2023) emphasise the need for enhanced training for school professionals and sustainable funding to strengthen school-based mental health services. Anttila et al. (2023) advocate for integrating mental health promotion within school health care to reduce stigma and increase accessibility.

In high-pressure educational systems, such as in Hong Kong, the urgency for mental health support is even greater (Hong Kong Federation of Youth Groups, 2024; The Standard, 2024). Driven by the prevalence of anxiety and depression among students (Shek et al., 2022), there has been a significant rise in mental health cases in schools post-COVID-19 (Kerr et al., 2021) and consequently a greater need for emotional support. While schools have begun exploring technology-assisted solutions to fill gaps in mental health services and prioritise early intervention, traditional methods often fall short in addressing the varied and culturally specific needs of students, such as refugee students (Baak et al., 2020). While AI chatbots offer potential benefits, questions remain about their adequacy and reliability compared to human therapists.

### 1.2. Current Applications, Benefits, and Limitations of AI in School Mental Health Services

AI-powered chatbots like Woebot (Fitzpatrick et al., 2017) and Wysa (Inkster et al., 2018) have emerged as tools that can provide scalable mental health support. These chatbots use natural language processing and machine learning to engage students in cognitive–behavioural therapy (CBT) techniques and other interventions (Fulmer et al., 2018). Morrow et al. (2023) found that users reported reduced feelings of loneliness and increased perceived support after using these platforms. The benefits of AI chatbots include accessibility, cost-effectiveness, and 24/7 availability, making them valuable for educational institutions facing resource constraints (D’Alfonso et al., 2017; Inkster et al., 2018).

However, limitations remain. Inkster et al. (2018) highlight that while AI can mimic certain therapeutic techniques, it often lacks the emotional depth and distinct understanding that human therapists provide. The “therapeutic misconception”, as discussed by Khawaja and Bélisle-Pipon (2023), refers to a phenomenon where users overestimate AI’s capabilities, potentially leading to misplaced trust, unmet needs, and underuse of human-provided therapy (Ciechanowski et al., 2019). The concept of therapeutic misconception originated from clinical trial settings to describe participants’ mistaken belief that the primary purpose of an intervention is to benefit them personally rather than as an experimental tool. In the context of artificial intelligence (AI), it refers to a misunderstanding or overestimation by users about what AI tools, particularly those designed for mental health and therapy, can actually provide in terms of therapeutic support. Furthermore, studies such as those by Zhang and Wang (2024) and Egan et al. (2024) caution that without genuine empathy, the perceived value of AI interventions may be limited, especially in addressing severe mental health conditions.

Research has shown that students generally hold a positive perception of AI chatbots with an awareness of their shortcomings. As evidenced by Moldt et al. (2023), medical students expressed trust in chatbots for supportive tasks such as answering questions and providing basic information, while remaining cautious about their use in more sensitive areas like personal counselling. This sentiment is echoed in the work of Kretzschmar et al. (2019), which highlights young people’s ethical perspectives on using automated conversational agents for mental health support. This study emphasises the importance of privacy, efficacy, and safety in the deployment of these technologies, suggesting that while students may appreciate the convenience of chatbots, they also harbour concerns regarding their limitations.

Several studies explored the effectiveness and feasibility of AI-based interventions in improving university students’ mental health. For example, Fitzpatrick et al.’s (2017) randomised controlled trial compared a group of 34 college students engaging with a conversational agent called Woebot and 36 college students using an ebook to manage their depression and anxiety. After two weeks, the Woebot group displayed lessened anxiety and depression symptoms. While the participants expressed their liking for the bot’s empathy and its ability to facilitate learning, they also reported negative experiences such as the bot not being able to understand certain user responses or react to unanticipated answers, repetitive answers, and technical problems. He et al.’s (2022) study was conducted with 148 college students in China, who were assigned to three groups: 49 used XiaoE, a mental health chatbot that was capable of fully automatic intelligent interaction with users; 49 read an e-book on depression; and 50 used Xiaoai, a general chatbot not designed for mental health services. Compared to e-book and general chatbots, XiaoE displayed significant short-term and long-term effectiveness in reducing users’ depression. However, participant feedback on the worst experience with XiaoE included repetitive and mechanical responses, rigid and tedious interactions, and technical glitches.

Klos et al. (2021), on the other hand, reported no significant difference between a group that used an AI-based chatbot and a group that read an electronic psychoeducation book for anxiety and depression, and some of their participants highlighted that the AI-based chatbot provided inaccurate responses to user input. Other challenges of AI therapists that have been reported in research include concerns about cybersecurity and their inability to empathise or assist with complex mental issues (Gbollie et al., 2023); as well as lack of sensitivity to users’ emotional needs and interactions that are too formal (Kuhail et al., 2024). As these studies were focused on tertiary students, the findings may not be generalisable to secondary school students.

In contrast, there is relatively limited research on the use of AI therapists among secondary school students. De Nieva et al. (2020) investigated senior high school students’ use of Woebot to manage academic stress. After two weeks of daily sessions with Woebot, the participants demonstrated decreased stress level. Among the weaknesses of Woebot stated by the participants were inability to understand user inputs, irrelevant responses, and absence of human-like interaction. In a study conducted by Fung et al. (2023) in Hong Kong, secondary school students received counselling services from a chatbot on academic and personal matters. The participants generally found the chatbot useful but also commented that the responses were too mechanical to offer personalised advice. As emphasised by the primary care providers in Nicol et al.’s (2022) research, chatbot therapists can help fill the gap while patients are on the waiting list to obtain an appointment with a human therapist, but they are incapable of providing the type of close clinical monitoring needed for adolescents with suicide and self-harm tendencies.

A review of current studies shows that existing research tends to focus on tertiary students. Since the academic and emotional issues adolescents face differ greatly from those of tertiary students, the findings may not be applicable in secondary school settings. Further, secondary school students are faced with increased risks for mental health problems brought about by academic pressure (Steare et al., 2023) and puberty (Patton & Viner, 2007). With a dearth of research into AI mental health therapy for adolescents, there is a need to explore secondary school students’ experience and perceptions of the use of AI therapy, such as GenAI chatbots, in particular the challenges they face, so that measures can be taken to optimise the potential of AI tools as mental health therapists.

### 1.3. The Three Challenges of the Digital Therapist Framework

Grodniewicz and Hohol (2023) outline the key obstacles AI faces in replicating human psychotherapy. They derived a conceptual framework by conducting a comprehensive literature review and analysing current AI applications in mental health. They examined case studies of existing AI chatbots like Woebot to identify how these systems simulate psychotherapeutic interactions and where they fall short. Their study outlines three key challenges for AI in psychotherapy: the Problem of a Confused Therapist, the Problem of a Non-human Therapist, and the Problem of a Narrowly Intelligent Therapist. This multi-source analysis allowed them to construct a framework that highlights significant barriers to AI effectively delivering psychotherapy.

**The Problem of a Confused Therapist.** Grodniewicz and Hohol (2023) argue that the field of psychotherapy is not monolithic but instead consists of many different therapeutic traditions and approaches. As estimated by Prochaska and Norcross (2018), there are now more than 500 different psychotherapeutic approaches, demonstrating the field’s diversity and sometimes conflicting nature. This variety makes it difficult to create a singular AI system capable of effectively applying diverse psychotherapeutic techniques. The challenge lies in understanding which processes and interventions are essential for effective psychotherapy, a question that remains unresolved even among human therapists. In addition, the psychoanalytic and cognitive–behavioural paradigms in mental health present contrasting conceptualisation and approaches to therapies (Milton, 2001). These problems highlight the need for clarity in determining the foundational components of effective psychotherapy for AI training (Rosenzweig, 1936; Weinberger, 1995).

**The Problem of a Non-human Therapist.** One of the most significant challenges discussed is whether AI can replicate the human-centric aspect of therapy. Traditional psychotherapy relies heavily on the interpersonal relationship between the therapist and the patient, which involves empathy, trust, and mutual understanding (Norcross, 1990). While AI can mimic some elements of human interaction, such as agreeing on tasks and goals, it struggles to form deep relational bonds (Grodniewicz & Hohol, 2023). Chatbots may be experienced as agents, but they lack the true capacity for human interaction characterised by discursive practices, understanding, and insight, thus limiting their role to that of tools rather than full-fledged therapists (Sedlakova & Trachsel, 2022). Kaveladze and Schueller (2023) argue that many successful therapeutic outcomes depend on the quality of the therapist–patient relationship, which current AI systems cannot replicate fully. According to Bordin’s (1979) therapeutic alliance model, the therapist–patient partnership consists of three interrelated components, namely, agreement on goals, agreement on tasks, and bond formation, encompassing both cognitive and affective aspects of therapy. Such complex interactions may not be easily emulated by AI, as AI tends to lack the capability to detect and respond to subtle emotional cues (Sedlakova & Trachsel, 2022) and sustain deep, meaningful conversations (Tekin, 2023). Due to these shortcomings, Lederman and D’Alfonso (2021) have called for a redefinition of the concept of therapeutic alliance within the digital context.

**The Problem of a Narrowly Intelligent Therapist.** Grodniewicz and Hohol (2023) point out that current AI systems are examples of narrow AI, which excel at specific, well-defined tasks but are not equipped to handle the adaptable and holistic nature of human psychotherapy. Examples such as IBM’s Deep Blue and AlphaGo illustrate the success of artificial narrow intelligence (ANI) in complex, rule-based environments like chess and Go (Legg & Hutter, 2007). These systems utilise deep reinforcement learning and neural networks to optimise performance in a constrained context. However, these advancements do not translate to the adaptive intelligence needed for psychotherapy, which involves responding fluidly to a wide range of scenarios. The therapeutic process often requires navigating unpredictable dialogues and emotional responses—elements that ANI cannot fully manage because it relies on pre-set programming and is unable to adapt to unpredictable scenarios (Floridi, 2023; Pandey et al., 2022). Until the development of artificial general intelligence (AGI), fully fledged AI-based psychotherapy may remain out of reach (Altman, 2023; Ullman, 2023). This study uses Grodniewicz and Hohol’s (2023) framework as the conceptual basis for analysing secondary school students’ perceptions of AI chatbots as therapists. This framework was chosen due to its suitability for the purpose of this study to explore the challenges of using AI chatbots as therapists. There are very few existing frameworks for understanding AI use in therapy. The most frequently employed frameworks in current research are ethical-based, such as those demonstrated in Fiske et al.’s (2019), Vilaza and McCashin’s (2021), and Khawaja and Bélisle-Pipon’s (2023) studies. Although Bordin’s (1979) therapeutic alliance model can be used to explain the complex relationship between the therapist and the patient, it originated from human-to-human therapeutic contexts, and thus it may not fully capture the challenges of engaging non-human therapists. The framework used in this study, based on Grodniewicz and Hohol’s (2023) three challenges of AI therapists with supporting literature from the field, is summarised in Table 1.

### 1.4. The Present Study

The objective of this study was to explore the challenges secondary school students experienced with the use of GenAI chatbots as mental health therapists using Grodniewicz and Hohol’s (2023) digital therapist framework. This study was driven by a main research question: What are the challenges of using GenAI chatbots as therapeutic tools from the perspective of secondary school students? By utilising systematically collected student reflections, this empirical research aimed to bridge theoretical perspectives with practical insights to enhance the understanding of the challenges AI must address to become a viable tool for school-based psychotherapy. The Introduction section provides the background of the research topic, identifying the gap in existing research and justifying the purpose of this study. In the next section, the research approach and data analysis procedure are explained, followed by an explanation of the findings in Section 3. Section 4 is an in-depth discussion of the findings and their implications for AI developers, policy makers, school administrators, and counsellors. Finally, the concluding section summarises the key findings, identifies the limitations of this study, and offers suggestions for future research.

## 2. Methodology

This study employed an interpretative qualitative research approach to investigate secondary school students’ perceptions of using AI chatbots as mental health therapists. Interpretative research examines the “what”, “how”, and “why” of a topic (Wiesner, 2022), and thus it is suitable for the purpose of this study to examine the challenges of using AI therapists in secondary school settings. The data were collected from an exercise within an AI literacy course organised by a university in Hong Kong. The course was hosted by the university, and students participated online from various locations. The course consisted of 10 h of online modules, which included hands-on practice and covered topics such as basic AI terminologies, prompt engineering, AI ethics, and the development of AI-integrated assessments with a focus on student partnership. Participants were given opportunities to explore various GenAI technologies for tasks like image generation, video creation, and text editing to gain familiarity with GenAI’s multimodal capabilities and achieve an intermediate level of AI literacy. As part of the course, students also built a simple chatbot. Students had one year to engage with chatbots and the course.

The main exercise posed the question, “Would you open up to an AI chatbot therapist?” Students were encouraged to elaborate on their responses to provide comprehensive insights into their thoughts, feelings, and concerns about AI’s role in a therapeutic setting. Participants also engaged with AI chatbots, such as GPT-4, to assess their responsiveness to emotional prompts and reflect on their interactions.

A total of 69 students from various local secondary schools in Hong Kong, ranging in age from 13 to 17 years (mean age: 15), participated in this study, with 35 females, 21 males and 13 who did not indicate gender. Convenient sampling was adopted as participation in this study was on a voluntary basis. Since the data were collected during an AI literacy course, convenience sampling was deemed appropriate because the participants were readily available (Etikan et al., 2016). Due to this reason, the participants were a homogeneous group sharing similar cultural and social backgrounds. All participants gave their informed consent before participating in this study. Sixty-nine written responses were collected from the participants, and they were anonymised before the analysis. Ethical approval for this study was obtained before data collection.

### Data Analysis

This study employed a deductive thematic analysis, using the conceptual framework of Grodniewicz and Hohol’s (2023) three challenges in AI psychotherapy as a general guide (see Table 1): the Problem of a Confused Therapist, the Problem of a Non-human Therapist, and the Problem of a Narrowly Intelligent Therapist. The data collected from student responses were first reviewed to ensure familiarity. Initial coding was conducted with these pre-established themes in mind by classifying phrases in student reflections according to the three challenges of AI therapy. Within each theme, similar codes were grouped together to form subthemes that further described the theme.

The analysis process was structured but flexible, as outlined by Hsieh and Shannon (2005), allowing for the recognition of emergent subthemes that extended beyond the initial framework. If there were new insights or challenges that did not align with the three existing themes, they were noted and incorporated through an inductive approach. This ensured that while the analysis was framed by existing theoretical insights, it remained open to novel findings, providing a comprehensive understanding of students’ perceptions of AI as a therapeutic tool.

The analysis was performed manually by the author. To enhance trustworthiness of the analysis, the author invited another researcher to review and confirm the coding, themes, and subthemes generated by the author. Through discussion with the invited reviewer, the author revised part of the coding and the additional themes that were not covered by Grodniewicz and Hohol’s framework. The findings are presented in the following section.

## 3. Findings

The findings from this study provide insight into secondary school students’ perceptions of AI as a potential chatbot therapist using Grodniewicz and Hohol’s (2023) conceptual framework. The analysis, supported by qualitative data from open-ended student responses, reveals the complex challenges and potential of AI chatbots as therapeutic tools. The data identified the main challenges across three primary areas: the Problem of a Confused Therapist, the Problem of a Non-human Therapist, and the Problem of a Narrowly Intelligent Therapist. The analysis also identified 11 subthemes that further describe Grodniewicz and Hohol’s (2023) three challenges and three additional themes that are not covered by their framework (see Table 2). A total of 147 codes emerged from the student responses, capturing a broad range of perspectives on the viability and limitations of AI in therapeutic settings.

### 3.1. The Problem of a Confused Therapist

This overarching challenge, highlighted by a total of nine responses, focuses on AI’s struggle to encompass the diverse and multifaceted nature of psychotherapy, maintain consistency, and apply varied techniques effectively.

**Inability to Reconcile Diverse Psychotherapeutic Approaches.** This subtheme, highlighted in three responses, shows a limited yet important awareness of AI’s challenges in covering the diverse range of therapeutic approaches. Student 42 remarked, “There are different aspects of therapy, like support, guidance, insight, goal setting… I believe that ChatGPT can become a therapist but should not be the only option”. This suggests that while AI can handle some aspects of therapy, its ability to adapt to various therapeutic traditions is uncertain, aligning with the findings by Prochaska and Norcross (2018). Grodniewicz and Hohol (2023) emphasise that this diversity is vital for effective psychotherapy, which AI currently struggles to achieve. Student 3 noted, “AI applications are less subject to bias than authentic humans… capable of analysing people’s woes and personal troubles from an objective perspective”, recognising AI’s objectivity but hinting that this may come at the cost of personalised and adaptive therapy. Student 63 shared an experience that reflects AI’s inability to provide analytical emotional support for effective therapy: “AI… was telling me how to be happy and how to get out from sadness… Whatever I said, it would only give me positive replies and advice. That’s not what I actually need!”

**Difficulty in Defining Core Components.** This subtheme was noted in four responses. Student 3 commented, “At the end of the day, AI is still a relatively new field of technology, and much of its capabilities remain largely unexplored”, which aligns with Grodniewicz and Hohol’s assertion that defining core therapeutic components for AI remains elusive. Student 38 observed, “AI can make the safest decision for the patient, but a real doctor could also focus on the feeling and the willingness of the patient”, pointing out AI’s challenge in integrating core, human-centred aspects of therapy, such as empathy and patient-centred decision-making. Student 12 expressed concern about relying on AI for mental health support: “We should never take the risk of people’s mental health”. This suggests an underlying uncertainty about whether AI can fulfil essential components of effective therapy. The data underscore the tension between AI’s algorithmic approach and the need for deeply humanistic elements integral to psychotherapy.

**Inconsistency in Application of Techniques.** Three students mentioned this issue, highlighting AI’s tendency to produce inconsistent responses. Student 9 reflected, “Although AI tends to be nice to humans, they are still not yet fully developed to understand commands properly. During my interactions with AI during the course, the AI would sometimes answer in a different language”. This comment highlights the variability in AI outputs that can compromise its reliability in therapy. Similarly, Student 63 added, “ChatGPT is just an emotionless existence… Sometimes we need not only advice, we also need hugs! At that moment, ChatGPT would be useless”. Student 8 raised concerns about AI’s ability to grasp complex contexts: “AI bots cannot fully understand the depth and implications of certain situations, especially if they’re complicated and involve grey areas”.

### 3.2. The Problem of a Non-Human Therapist

This theme was the most prevalent in student responses with 91 mentions, signifying a profound concern regarding AI’s inability to replicate human empathy, build trust, understand emotional cues, and form deep connections.

**Lack of Empathy and Genuine Human Connection.** Empathy emerged as a prominent concern, with 29 references underscoring this issue. Student 37 highlighted, “I do not believe that communicating with an AI is the same as communicating with a human… it is just a bunch of cold words generated by an AI”, pointing out the perceived lack of warmth and depth in AI interactions. Student 50 further emphasised this by stating, “While I do believe that chatbots can make good therapists, I do think there is a flaw to it… the human connection is an extremely complex and powerful thing that is only limited to humans and can only be achieved through physical contact”. These reflections resonate with Grodniewicz and Hohol’s (2023) perspective that empathy—critical to effective therapy—cannot be authentically replicated by current AI systems.

Some student responses expressed strong opposition to using generative AI for therapeutic purposes. Concerns were raised about trust and security: “I believe most people would also be reluctant to share their real emotions while knowing the dangers of AI” and “ChatGPT is not meant for therapy purposes, nor has it been tested for its effectiveness in therapy”. These insights collectively highlight that while AI can provide certain objective insights, it struggles to deliver the empathetic, human-centred approach that many deem essential for effective therapy.

**Challenges in Building Trust and Therapeutic Alliance.** Twenty-five responses highlighted lack of trust as a barrier. Student 54 remarked, “Miscommunication is a risk, as chatbots may misinterpret user inputs, potentially offering unhelpful advice”, pointing to the technological limitations that impede trust-building. Student 4 indicated a distrust of AI’s use in therapy due to the potential for misuse and manipulation. It reflects the student’s concern that AI could be exploited, hindering the establishment of trust essential for therapeutic relationships: “AI might be controlled by other people with bad intentions, and they would use this ‘therapist’ to control and manipulate human beings’ emotions, creating fake information and may bring chaos to the society”. Student 11’s insight, “Some people may also feel paranoid about their information being leaked… AI is still a computer and a machine”, underscores data privacy concerns that affect trust. While Grodniewicz and Hohol emphasise the importance of trust in successful therapy, the data reveal that students remain wary of whether AI can offer the confidentiality and personalised engagement required to foster it. Student 16 added, “However, I’m not sure that revealing your personal experiences to an AI is safe… There are currently no laws against AI exploiting human needs and emotions”, pointing to regulatory gaps that further weaken trust. In Torous and Blease’s (2024) editorial piece, they also highlighted that all major AI chatbot companies currently state that their products must not be used for clinical purposes. Student 66 noted that AI’s avoidance or inadequate response to sensitive topics undermines trust: “When people talk about sensitive topics such as suicide, AI will avoid the topic and doesn’t really answer you”.

Several students voiced apprehensions that go beyond general ethical considerations to highlight specific fears regarding privacy and the potential for harmful control. Student 4’s statement underscores this concern: “Who knows what ChatGPT will do with our information and feelings?” This showcases a deep-seated anxiety about data privacy and potential exploitation, reflecting how the ethical and safety implications of AI interactions continue to be a significant barrier to trust in digital therapy.

**Limitations in Understanding Emotional Cues.** Eighteen responses touched upon AI’s difficulty in understanding and responding to emotional cues. Student 4 expressed doubt about AI’s current ability to identify and interpret human emotions accurately, limiting its role as an effective therapeutic tool. This reflects broader concerns about AI’s capacity to process emotional subtleties. Student 61 stated, “I would not open up to a chatbot therapist because I feel like it cannot really understand what emotions I am having”, emphasising this limitation. Student 65 supported this view, noting, “AI is limited to understanding expressions based on what has been programmed… may assume a response… not accurate or sensible”. This corresponds with the literature that describes AI’s challenges in interpreting emotions beyond programmed responses (Floridi, 2023; Pandey et al., 2022).

The recognition of emotional cues is essential for therapy, as it is integral to creating meaningful and effective interactions. Student 41 pointed out AI’s inability to detect hidden emotions or deeper meanings in conversation, asking, “Can they read between the lines? Can they correctly identify the hidden emotions that are beyond what the person types?” These perspectives emphasise that while AI can provide structured responses, its inability to fully grasp human emotions limits its potential as a reliable therapeutic tool.

**Absence of Deep Relational Engagement.** Nineteen students emphasised that AI fails to deliver deep, meaningful relationships essential for therapy. Student 57 explained, “The familiarity and emotional closeness that come from interacting with a human can encourage individuals to share more openly. A chatbot cannot replicate that sense of intimacy”, emphasising that trust and intimacy are closely tied to human interaction. Student 50 shared, “For me, the reason for the success of traditional therapists is the understanding between the patient and the therapist themselves, and chatbots as therapists just take this unique feature away”, pointing out that relational depth is fundamental for a therapeutic alliance, a view supported by Grodniewicz and Hohol’s framework. Student 37 stated, “Although AI could recognise our emotions through our punctuation and sentence structure, I do not believe that communicating with an AI is the same as communicating with a human”. This response underscores that while AI can recognise and respond to user input through natural language processing on a basic level, it cannot foster deep relational engagement that humans require for effective therapy, suggesting limitations in long-term therapeutic relationships.

Another limitation of AI therapists is the inability to offer contextualised advice and a lack of shared understanding. Student 41 articulated this well, noting, “What can an AI do? If the person types out excuse after excuse, how can we expect an AI to be able to grasp what they actually feel if it’s just words they are analysing? They may be able to give some words of comfort, but they would be awfully general and not the personal type of reassurance a person who goes into therapy wishes to seek from their therapist”. This insight demonstrates how shared understanding and real-life empathy play a crucial role in building a therapeutic alliance, something AI struggles to emulate.

### 3.3. The Problem of a Narrowly Intelligent Therapist

This category was represented by 41 student responses, which focused on the limitations of AI’s specialised task capabilities and adaptability.

**Narrow AI’s Limited Task Specialisation.** This subtheme was evident in student responses pointing to AI’s limited ability to engage in complex, emotionally attuned therapy. Student 1 remarked, “AI doesn’t do a great job of understanding human emotions. With AI being what it is right now, I would refrain from using it as my therapist”. This view suggests that AI struggles to match the multifaceted and adaptive responses needed in therapeutic contexts.

Similarly, Student 44 stated, “But I don’t really think the AI we have now can give a great suggestion to people or comfort them, because AI still cannot understand human emotions”. These responses underscore the perception that AI’s task specialisation, though potentially useful for basic guidance and structured responses, is inadequate when it comes to the deeper, more adaptive aspects of emotional and psychological support. The narrow focus of AI systems limits their ability to provide the kind of empathetic, personalised care required in therapeutic settings, confirming the concerns within the subtheme of limited task specialisation.

**Dependence on Pre-programmed Responses.** Twenty-three mentions supported this subtheme. Student feedback highlighted that AI often relies on pre-programmed responses, lacking genuine understanding. One student shared, “Although AI could recognise our emotions through punctuation and sentence structure, it cannot provide the comfort I would hope to seek; it is just a bunch of cold words generated by an AI”. Student 61 explained, “AI interactions lack the emotional depth needed to process emotions beyond predefined parameters”, echoing the literature by Legg and Hutter (2007) on ANI’s success in structured, rule-based tasks versus the dynamic nature of human interaction in therapy. Legg and Hutter (2007) discussed the success of ANI in specific, well-defined tasks like chess and Go; however, they specified that such structured environments are vastly different from the dynamic nature of human interaction in therapy. This contrast was noted by a student who explained, “AI is limited to understanding expressions based on what has been programmed; it will be completely clueless about other complex emotions or may assume a response for a certain situation which may not be accurate or sensible”.

**Inability to Adapt to Complex, Unpredictable Human Behaviours.** This subtheme emerged in 14 student responses. The risk of AI misinterpreting inputs and responding inappropriately was a recurring concern, highlighting its limited adaptability. One student expressed, “There is always a chance that the bot will say the wrong thing and make matters worse. Each person is different and might want to hear different things; a chatbot would not be able to adjust to different people’s responses”. Another mentioned, “There will still be a chance of using harmful words… if the AI uses harmful words, it will make you even more anxious and difficult to handle the matter”. Several students also noted that AI struggles with handling complicated situations. The consistency and automatic nature of AI responses were cited as problematic, with one student saying, “ChatGPT’s responses are consistent, which feels fake and automatic to me, lacking empathy”. This highlights that while AI may provide technically correct responses, they lack the flexibility needed to navigate complex human conversations. Similarly, another student observed, “AI therapists may give out false or discouraging information… the fact that it is artificial implies that it cannot understand all our thoughts and is prone to making mistakes”.

Lastly, there is the concern that AI’s inability to adapt can sometimes exacerbate problems rather than solve them. One student noted, “If the AI were to use harmful words, it would make you even more anxious… and may lead to inevitable consequences”.

**Lack of General Intelligence (AGI) Required for Comprehensive Therapy.** Two responses explicitly addressed this challenge, underlining a critical gap in AI’s current capabilities. Grodniewicz and Hohol (2023) contend that until AI progresses towards achieving AGI, its potential application in comprehensive therapeutic settings will remain constrained. This argument is mirrored in the perspectives shared by students. “To this day, we are unsure if AI is truly conscious and capable of processing emotions such as happiness, sadness, anger, and fear”. This reflection underscores the perceived inability of AI to engage deeply with emotional experiences or convey genuine consciousness. The absence of this level of awareness means that AI cannot replicate the true emotional engagement necessary for effective therapy, reinforcing Grodniewicz and Hohol’s (2023) assertion that the current AI paradigm falls short in delivering the comprehensive, emotionally attuned responses required for meaningful therapeutic interactions.

### 3.4. Emergent Themes Beyond the Framework

The analysis revealed three additional themes not encompassed within Grodniewicz and Hohol’s original framework.

**The Problem of an Objective but Detached Therapist.** This theme highlights the conflict between the non-judgemental, objective nature of AI feedback and its inability to provide true empathy and personal connection. In contrast to The Problem of a Non-human Therapist, which explains the negative characteristic of AI lacking empathy, this theme describes a more nuanced perspective of favoured objectivity and the unfavoured detached nature of AI that comes with objectivity. Some students appreciated the non-biased, judgement-free nature of AI, finding solace in its impartiality. Student 13 captured this positive perspective by saying, “It is easier to open up to a chatbot because sometimes people feel they are bothering others when discussing issues. Chatting with a chatbot doesn’t bother anyone”. However, others pointed out that this objectivity came at the cost of true empathy, which is crucial for deeper emotional support. Student 37 expressed this clearly: “I believe it cannot provide the comfort I would hope to seek; rather, it is just a bunch of cold words generated by an AI”. This dichotomy points to an important balance that AI therapy must strike between offering non-judgemental support and maintaining the human touch that fosters emotional connection.

**The Problem of a Dependence-Inducing Therapist.** This theme focuses on the risk of developing unrealistic expectations or reliance on AI therapy, which may hinder individuals from seeking real professional help. Students pointed out that overreliance on AI could lead to unrealistic expectations of digital therapeutic support, potentially delaying or preventing individuals from seeking real, professional help when needed. Student 21 noted this potential pitfall by saying, “Users may overestimate the therapeutic benefits and underestimate the limitations of using such technologies, further deteriorating their mental health. Such a phenomenon can be classified as a therapeutic misconception where users may infer the chatbot’s purpose is to provide them with real therapeutic care”. This concern highlights the need for clear boundaries and public education around the role of AI in mental health to prevent over-dependence on its capabilities, and this finding echoes with the discussion on therapeutic misconceptions by Khawaja and Bélisle-Pipon (2023).

**The Problem of an Anonymous but Impersonal Therapist.** This theme explores the trade-off between the comfort provided by the anonymity of AI and the absence of genuine human connection and emotional engagement. Students found value in the anonymity AI therapy provides, which allows them to open up without fear of stigma. Student 25 shared this perspective, saying, “The anonymity of ChatGPT therapy is also attractive. It can provide a sense of security and comfort. I think this anonymity allows us to explore our emotions without fear of judgement or shame from others”. However, this comes at the cost of authentic engagement, as AI lacks the ability to respond to subtle emotional cues and body language. Student 41 elaborated, “The reason why therapy sessions are always face to face is due to the fact that the body always betrays what the words don’t say; many physical cues will be missed if the person can select what to disclose to the AI without showing any weakness at all. That defeats the purpose of therapy”. This theme reflects the trade-off between the comfort of anonymity and the depth of human connection in therapy.

## 4. Discussion

The integration of generative AI (GenAI) into school-based mental health services introduces a complex dynamic that challenges traditional psychotherapy boundaries. This research focused on GenAI due to its enhanced capabilities over pre-GenAI iterations, marked by large language models, advanced functionalities, and the ability to engage in sophisticated dialogues. Anchored in Grodniewicz and Hohol’s (2023) framework, this study explored secondary school students’ perceptions of AI as a therapeutic tool. Participants, equipped with theoretical and practical knowledge from an AI literacy course, provided unique insights into the potential and significant limitations of AI in this sensitive context.

GenAI’s potential as a therapeutic agent in schools is supported by its technological advancements, including multimodal capabilities that allow it to “listen”, “see”, and process complex emotional cues, extending beyond simple text analysis. Students’ familiarity with these tools enabled them to critically assess AI therapy, offering perspectives that align with and extend existing literature. Obtaining empirical data on mental health services from school students can be challenging, as such services need to be treated with care. The data collected are sensitive, and sample sizes are often small as only a limited number of students have experience with such services. However, while this study did not involve direct mental health services, the students’ enhanced literacy in GenAI enabled them to provide sufficient and accurate responses regarding their perceptions of using GenAI as therapists in a school setting.

### 4.1. Perception of AI’s Versatility in Therapy: The Problem of a Confused Therapist

Central to the findings was the perception of AI’s ability to adapt to the multifaceted nature of psychotherapy. Grodniewicz and Hohol (2023) highlight the “Problem of a Confused Therapist”, and the analysis revealed three specific issues associated with this challenge: inability to reconcile diverse psychotherapeutic approaches, difficulties in defining core components, and inconsistency in application of techniques. These issues resonated in student responses, where doubts were raised about AI’s adaptability. Student 42 noted that while AI could offer basic guidance and structured advice, it lacked the comprehensive understanding needed for individual therapeutic needs. As pointed out by the participants in Fitzpatrick et al.’s (2017) study with college students, chatbots are unable to understand complex user inputs and incapable of responding to unexpected answers. This lack of versatility sometimes results in inaccurate and irrelevant responses, as reported by previous studies (De Nieva et al., 2020; Klos et al., 2021). These issues align with Prochaska and Norcross’s (2018) assertion that psychotherapy encompasses a broad range of approaches. The synthesis of these approaches into a coherent AI model remains exceedingly complex.

Moreover, some students noted AI’s objectivity as a double-edged sword—capable of unbiased analysis but at the cost of personalised emotional engagement. This observation is consistent with existing research (Ciechanowski et al., 2019), which cautions that reliance on AI’s algorithmic processes may lead to overgeneralised responses that do not account for individual nuances.

### 4.2. Trust and Emotional Connection: The Problem of a Non-Human Therapist

The most profound theme identified was the “Problem of a Non-human Therapist”, which is further described by four subthemes: lack of empathy and genuine human connection, challenges in building trust and therapeutic alliance, limitations in understanding emotional cues, and absence of deep relational engagement. The literature supports this finding, with Norcross (1990) and Sedlakova and Trachsel (2022) underlining that empathy and genuine human connection are core to successful therapy. The students’ reflections revealed that despite GenAI’s capability to simulate conversations, it often lacks the depth required to build trust and establish emotional bonds. This concern is evident in Student 37’s statement that AI responses felt like “a bunch of cold words”, a sentiment echoing Grodniewicz and Hohol’s (2023) identification of AI’s limitations in replicating human-centric therapeutic relationships. These findings echo the issues identified by Gbollie et al. (2023) and Kuhail et al. (2024) in their studies.

Concerns about data privacy and potential misuse of information emerged as barriers to trust, reflecting broader ethical considerations in AI use. This concern aligns with Torous and Blease’s (2024) work on the regulatory vacuum surrounding AI applications in therapy. Students expressed hesitance to share personal information, fearing data vulnerability and exploitation, which underscores the importance of stringent data protection measures. Similar challenges were also reported by Gbollie et al. (2023), who found data security and privacy to be a major concern among users of digital mental health solutions.

### 4.3. Adaptability and Comprehensiveness: The Problem of a Narrowly Intelligent Therapist

Grodniewicz and Hohol’s (2023) third challenge, the “Problem of a Narrowly Intelligent Therapist”, is crucial in understanding students’ perceptions of AI’s role in therapy. The findings of this study provide evidence for four specific issues concerning this challenge: narrow AI’s limited task specialisation; dependence on pre-programmed responses; inability to adapt to complex, unpredictable human behaviours; and lack of artificial general intelligence (AGI) required for comprehensive therapy. Despite GenAI’s enhanced multimodal capabilities, which theoretically allow for a more sophisticated understanding of user inputs, students reported limitations in its ability to adapt to complex and unpredictable scenarios. For example, Student 44 noted that while GenAI might offer structured support, it falls short in situations that require emotional insight or a personalised approach.

The literature on narrow AI’s performance in specialised tasks supports these observations, emphasising that while ANI can excel in structured, rule-based interactions, its lack of general intelligence impedes its utility in comprehensive therapeutic settings. Students’ reflections revealed that AI’s pre-programmed responses often failed to resonate, lacking the flexibility needed to address unique emotional states effectively. The problem with mechanical, pre-programmed responses has been widely reported in previous research. For example, the participants in He et al.’s (2022) study criticised the AI-based chatbot’s responses as rigid, resulting in tedious interaction. In addition, Fung et al.’s (2023) research shows that mechanical responses contributed to secondary school students’ perception that AI chatbots were incapable of offering personalised advice. This limitation also aligns with the findings of Egan et al. (2024), which emphasise the risk of AI’s inability to adapt to evolving therapeutic dialogues, potentially leading to user frustration or disengagement.

### 4.4. Emerging Perspectives

The three emergent themes discussed in the analysis reveal a complex duality that students familiar with GenAI are beginning to realise. Each theme embodies both positive and negative aspects that present a balance or tension in their potential application in therapy.

The first theme, the Problem of an Objective but Detached Therapist, highlights the duality between AI’s non-judgemental, objective feedback and its inability to provide genuine empathy. On the positive side, students appreciated that AI could offer impartial, judgement-free responses that made them feel comfortable discussing issues that might otherwise carry stigma or embarrassment. This objectivity can create a space where students feel free to express themselves without fear of bias. However, the downside is that this very objectivity can lead to perceptions of coldness or detachment. AI’s inability to provide authentic emotional engagement means that while it may respond logically or offer structured advice, it cannot replicate the warmth and connection of a human therapist. This dichotomy highlights the challenge in balancing AI’s strength in impartial analysis with the need for an empathetic response that resonates on a human level. This shortcoming affirms Nicol et al.’s (2022) assertion that AI therapists are unsuitable for adolescents who need close clinical monitoring for mental health issues.

The second theme, the Problem of a Dependence-Inducing Therapist, underscores the potential of AI to foster overreliance, which could deter students from seeking real professional help. The positive aspect is that AI can be a readily available, accessible support system, providing students with immediate answers or guidance during moments of distress. This is especially valuable in environments where human resources are limited. However, the risk lies in users developing unrealistic expectations of AI’s capabilities, leading to therapeutic misconceptions. The traditional boundaries between the therapist and the client in in-person counselling are blurred in AI-enhanced, as advice and guidance can be obtained on demand, distorting the therapeutic frame (Vagwala & Asher, 2023). As Gray (1994) asserts, by not making themselves available 24/7, therapists allow clients to learn to handle the issues they face independently. Hence, removing the wait time may work against clients’ mental health rather than for it. In addition, students may begin to perceive AI as a full substitute for human therapy, potentially resulting in unmet needs or even a deterioration of mental health when the AI cannot adequately respond to complex situations. Students’ reliance on AI can be further exacerbated by the personalisation features that enable it to offer advice and guidance tailored for clients’ needs (Kocaballi et al., 2019). This double-edged nature necessitates clear boundaries and education on the proper role of AI in mental health support.

The third theme, the Problem of an Anonymous but Impersonal Therapist, speaks to the trade-off between the comfort provided by AI’s anonymity and the lack of genuine human connection. On the positive side, anonymity can encourage students to open up more freely, discussing personal or sensitive topics without fear of judgement. This sense of security can lower barriers to seeking help and provide an initial layer of support. On the negative side, the impersonal nature of AI means that it cannot interpret subtle emotional cues or offer the nuanced support that comes with face-to-face interaction. This impersonal approach can leave students feeling unheard or misunderstood, highlighting the need to balance the benefits of anonymity with the irreplaceable value of human interaction. The challenges and limitations of AI therapists, as demonstrated by the key themes of this study, emphasise the notion of AI as a support, not as a substitute for human therapists (Fiske et al., 2019; Zhang & Wang, 2024).

### 4.5. The Role and Limitations of Direct User Feedback in Evaluating AI Therapy

User engagement and feedback play a crucial role in evaluating AI-based mental health interventions, yet they present inherent limitations when assessing the therapeutic effectiveness of chatbots. While direct user reflections offer valuable insights into the perceived usefulness of GenAI therapy, self-reported data are subjective and shaped by individual expectations, prior experiences with mental health support, and pre-existing biases toward AI (Kretzschmar et al., 2019). These factors influence how users interpret their interactions with chatbots, potentially leading to an overestimation or underestimation of AI’s capabilities.

Furthermore, the notion of “opening up” to an AI therapist carries theoretical complexity. In cognitive therapy, self-disclosure can facilitate cognitive restructuring, allowing individuals to articulate thoughts and challenge maladaptive thinking patterns. Emotion-focused therapy, however, considers disclosure as a process of exploring and validating emotions, which is essential for emotional healing (Elliott et al., 2004). AI chatbots, despite their ability to generate responses based on linguistic and contextual analysis, lack the emotional reciprocity and affective presence that characterise human-led therapy. This raises questions about whether AI-facilitated self-expression leads to meaningful psychological growth or if it remains a surface-level engagement.

Additionally, from a perceptual control theory perspective, therapeutic effectiveness is not solely about initial engagement but rather about prolonged, iterative exploration of issues that lead to deeper self-understanding and behavioural change. AI chatbots, such as MYLO, have attempted to integrate this model by encouraging users to re-engage with their issues across multiple interactions (Gaffney et al., 2020). However, without sustained emotional attunement, AI-driven therapy may struggle to replicate the long-term restructuring process that occurs in human-led interventions.

The assumption that engagement with AI equates to therapeutic effectiveness is also problematic. Previous studies suggest that users may interact with AI chatbots for reasons unrelated to emotional well-being, such as curiosity, convenience, or entertainment, which may distort assessments of their actual therapeutic value (Fitzpatrick et al., 2017). Furthermore, the risk of “therapeutic misconception” (Khawaja & Bélisle-Pipon, 2023) arises when users overestimate AI’s therapeutic potential, potentially leading to misplaced trust in digital solutions while underutilising professional mental health support.

To develop a more robust evaluation framework for AI-mediated therapy, future research should integrate multiple data sources, including behavioural metrics (e.g., frequency, duration, and depth of chatbot interactions), linguistic analyses of emotional expression, and psychometric assessments of well-being over time. By complementing direct user feedback with objective measures, a more comprehensive understanding of AI’s role in therapy can be established, ensuring that chatbot interventions are assessed not just by engagement levels but by their tangible impact on mental health outcomes.

### 4.6. The Future of AI in Therapy: Bridging the Gap

A compelling aspect of this research is the exploration of how future GenAI developments, with enhanced multimodal capabilities, might influence therapy. The potential of GenAI to incorporate visual and auditory inputs could theoretically bridge some gaps noted by students, such as its inability to read between the lines or detect non-verbal cues. If GenAI systems could analyse body language, facial expressions, and vocal tones, the problem of missing emotional cues might be mitigated. However, as this study suggests, even with these advancements, the depth of human empathy and the lived experiences that inform therapeutic interactions remain irreplaceable.

A key takeaway from this study is that while students recognise the potential of AI in supportive roles, significant challenges persist in achieving a balance between technological advancement and the emotional depth characteristic of human therapists. The notion that AI might one day integrate multimodal inputs to better emulate human-to-human interactions opens a fascinating line of inquiry but does not fully resolve concerns around trust and genuine empathy. This study contributes to a broader understanding that while GenAI can serve as an auxiliary tool in mental health settings, particularly in resource-constrained schools, it remains a supplement rather than a substitute for human connection.

### 4.7. Implications

The implications of this study extend across multiple dimensions, from the practical application of AI in school-based mental health services to the broader conversation about the role of technology in sensitive human interactions. One of the most compelling implications is the potential for AI to serve as a bridge in addressing the resource gaps prevalent in educational mental health services. Given the chronic shortages of qualified mental health professionals in schools (National Education Association, 2023), GenAI could act as a supplemental tool to provide immediate, preliminary support to students who might otherwise face significant delays before speaking with a human therapist. This could be particularly impactful in high-stress educational environments or regions where resources are scarce.

However, this study highlights that for AI to be genuinely effective, developers must prioritise advancing AI’s capabilities to simulate deeper empathy and build trust. Students’ concerns about AI feeling “cold” or lacking genuine emotional understanding underscore the necessity of integrating advanced natural language processing and machine learning techniques that can mimic human-like empathetic responses. The goal should not just be replicating therapeutic protocols but fostering interactions that students perceive as warm, trustworthy, and adaptive. This can involve designing AI systems that use multimodal inputs—such as analysing facial expressions and tone of voice—to better interpret emotional states and respond in a more contextually sensitive manner.

Educational institutions play a critical role in shaping how AI tools are implemented. Schools must adopt a balanced approach that acknowledges AI as a supplementary, rather than primary, source of mental health support. This implies that training for school counsellors and educators should include AI literacy, allowing them to understand the capabilities and limitations of these tools and guide students in using them responsibly. Moreover, establishing transparent and clear guidelines for AI’s role in mental health services can help manage expectations among students and reduce the potential for overreliance, which may lead to therapeutic misconceptions, as noted by Khawaja and Bélisle-Pipon (2023).

The ethical dimension of using AI for therapy is another significant implication. As student responses indicated, trust issues related to data privacy and security remain substantial barriers. This suggests that any AI implementation strategy must include rigorous data protection measures and comply with ethical standards that prioritise student safety. Building trust in AI tools will require not only technological enhancements but also policy frameworks that reinforce user confidence. Addressing these concerns transparently can make AI tools more acceptable and reliable in educational settings, ensuring that students feel secure in engaging with these resources.

The findings also prompt a re-evaluation of the potential for hybrid therapy models that blend AI support with human oversight. Such models could capitalise on AI’s strengths—such as 24/7 availability, cost-effectiveness, and scalability—while mitigating its weaknesses through human involvement. This approach could transform the accessibility of mental health support in schools, providing students with continuous monitoring and immediate feedback while maintaining the depth and adaptability that only human therapists can provide. Exploring these blended models could redefine the support landscape, offering a more holistic, layered approach to student mental health care.

Additionally, this study underscores the need for continuous improvement and user feedback mechanisms to ensure that AI remains adaptable and aligned with user needs. Educational institutions and developers should create spaces for ongoing dialogues with students and educators to refine these systems. By integrating iterative improvements based on real-world feedback, AI tools can evolve to meet the demands of mental health therapy more effectively.

Lastly, the results of this study could inform public policy and funding decisions aimed at enhancing school-based mental health services. Policymakers should consider investments in AI development and training programmes that equip schools with the necessary resources to incorporate AI responsibly. This includes not just financial support for technology but also comprehensive training programmes that enable educators and counsellors to maximise AI’s potential while understanding its boundaries. By fostering a culture of responsible AI use, schools can strike a balance between innovation and human-centred care, ensuring that students receive comprehensive and ethical mental health support.

## 5. Conclusions

The exploration of GenAI’s role in school-based mental health services underscores both the promise and challenges of integrating advanced AI tools into therapeutic settings. While GenAI offers unique benefits, such as accessibility and objectivity, its current limitations in fostering genuine empathy, building trust, and adapting to complex emotional scenarios highlight the need for careful implementation. This study, grounded in student perceptions gathered during an AI literacy course, illustrates that while students appreciate certain aspects of AI, they remain cautious about its capacity to replace human connection in therapy. The road ahead requires bridging technological potential with the irreplaceable qualities of human therapists to effectively support students’ mental health.

The use of Grodniewicz and Hohol’s framework, while insightful, presented challenges in analysis due to overlapping themes that sometimes blurred distinctions between categories. The interconnected nature of trust, adaptability, and empathy made discrete analysis complex, potentially limiting the clarity of subthemes. Despite these challenges, the framework provided a necessary structure to bridge theoretical discussions with empirical findings. Additionally, the demographic scope of secondary school students limits the generalisability of the findings across age groups and cultural contexts. As individuals’ perceptions are culturally and socially shaped, the issues and insights gained from this study may be relevant only to Chinese adolescents seeking digital mental health solutions in an Asian secondary school setting. The pre-existing AI literacy of participants, while beneficial for informed insights, might also have skewed perceptions, making them more critical or technically aware of AI’s capabilities and flaws; hence, the findings are more reflective of technology-literate individuals rather than the general student population. Another limitation concerns the participants’ engagement with ChatGPT-4—an LLM-based system as opposed to other more commonly used rule-based chatbots—in the AI literacy course, which might have influenced their perceptions and responses on therapeutic chatbots in this study. Finally, the qualitative nature of the research, while offering deep insights, also limits the ability to generalise findings broadly.

Future research should focus on longitudinal studies to track the sustained use of AI in therapy and observe shifts in student perceptions over time. Incorporating AI systems with multimodal capabilities, such as visual and auditory processing, may provide a more comprehensive understanding of whether these advancements can address current empathy and trust deficits. Additionally, hybrid models that blend human oversight with AI could be explored to evaluate how such combinations may enhance therapy’s effectiveness, ensuring that students receive holistic, supportive care. Future research should explore the perceptions and experiences of students with limited or no prior exposure to AI tools to obtain a comprehensive insight into the perceived potential of AI tools as virtual therapists among secondary school students. Furthermore, with the integration of quantitative approaches such as survey and structured observation, future research could explore the perceptions and user behaviour of a wider student population concerning AI therapy.

## Figures and Tables

**Table 1:** The three challenges of AI therapy identified by Grodniewicz and Hohol (2023).

Challenge	Description and Supporting Literature
**i. The Problem of a Confused Therapist**	A broad variety of psychotherapeutic methods (e.g., Prochaska & Norcross, 2018) Contrasting conceptualisations of mental health: psychoanalytic, cognitive–behavioural (Milton, 2001) Integrative therapeutic practices and the challenge of creating an AI that can mimic such complexity (Prochaska & Norcross, 2018)
**ii. The Problem of a Non-human Therapist**	AI lacks the capacity for true human interaction, limiting their role as therapists (Sedlakova & Trachsel, 2022) AI lacks depth in sustaining meaningful conversations over time (Tekin, 2023) AI therapists may not be able to emulate human therapists in relationship building (Kaveladze & Schueller, 2023) Therapeutic alliance needs to be redefined within the digital context (Lederman & D’Alfonso, 2021)
**iii. The Problem of a Narrowly Intelligent Therapist**	ANI’s (artificial narrow intelligence) specialisation in specific tasks in rule-based environments (Legg & Hutter, 2007) AI’s reliance on pre-set programming and limitations in improvisation (Floridi, 2023; Pandey et al., 2022) Current AI technologies may not be able to fulfil comprehensive therapy needs (Altman, 2023; Ullman, 2023)

**Table 2:** Themes and subthemes on the challenges of AI therapy.

Theme	Subtheme	Examples
**i. The Problem of a Confused Therapist**	Inability to reconcile diverse psychotherapeutic approaches	“AI applications are less subject to bias than authentic humans… capable of analysing people’s woes and personal troubles from an objective perspective.” (Student 3) “There are different aspects of therapy, like support, guidance, insight, goal setting… I believe that ChatGPT can become a therapist but should not be the only option.” (Student 42)
	Difficulty in defining core components	“At the end of the day, AI is still a relatively new field of technology, and much of its capabilities remain largely unexplored.” (Student 3) “AI can make the safest decision for the patient, but a real doctor could also focus on the feeling and the willingness of the patient.” (Student 38)
	Inconsistency in application of techniques	“Although AI tends to be nice to humans, they are still not yet fully developed to understand commands properly. During my interactions with AI during the course, the AI would sometimes answer in a different language.” (Student 9) “AI bots cannot fully understand the depth and implications of certain situations, especially if they’re complicated and involve grey areas.” (Student 8)
**ii. The Problem of a Non-human Therapist**	Lack of empathy and genuine human connection	“I do not believe that communicating with an AI is the same as communicating with a human… it is just a bunch of cold words generated by an AI.” (Student 37) “While I do believe that chatbots can make good therapists, I do think there is a flaw to it… the human connection is an extremely complex and powerful thing that is only limited to humans and can only be achieved through physical contact.” (Student 50)
	Challenges in building trust and therapeutic alliance	“AI might be controlled by other people with bad intentions, and they would use this ‘therapist’ to control and manipulate human beings’ emotions, creating fake information and may bring chaos to the society.” (Student 4) “When people talk about sensitive topics such as suicide, AI will avoid the topic and not really answer you.” (Student 66)
	Limitations in understanding emotional cues	“I would not open up to a chatbot therapist because I feel like it cannot really understand what emotions I am having.” (Student 61) “Can they read between the lines? Can they correctly identify the hidden emotions that are beyond what the person types?” (Student 41)
	Absence of deep relational engagement	“The familiarity and emotional closeness that come from interacting with a human can encourage individuals to share more openly. A chatbot cannot replicate that sense of intimacy.” (Student 57) “For me, the reason to the success of traditional therapists is the understanding between the patient and the therapist themselves, and chatbots as therapists just take this unique feature away.” (Student 50)
**iii. The Problem of a Narrowly Intelligent Therapist**	Narrow AI’s limited task specialisation	“AI doesn’t do a great job of understanding human emotions. With AI being what it is right now, I would refrain from using it as my therapist.” (Student 1) “But I don’t really think the AI we have now can give a great suggestion to people or comfort them, because AI still cannot understand human emotions.” (Student 44)
	Dependence on pre-programmed responses	“Although AI could recognise our emotions through punctuation and sentence structure, it cannot provide the comfort I would hope to seek; it is just a bunch of cold words generated by an AI.” (Student 37) “AI interactions lack the emotional depth needed to process emotions beyond predefined parameters.” (Student 61)
	Inability to adapt to complex, unpredictable human behaviours	“Each person is different and might want to hear different things; a chatbot would not be able to adjust to different people’s responses.” “There will still be a chance of using harmful words… if the AI uses harmful words, it will make you even more anxious and difficult to handle the matter.”
	Lack of artificial general intelligence (AGI) required for comprehensive therapy	“To this day, we are unsure if AI is truly conscious and capable of processing emotions such as happiness, sadness, anger, and fear.”
**iv. The Problem of an Objective but Detached Therapist**		“I believe it cannot provide the comfort I would hope to seek; rather it is just a bunch of cold words generated by an AI.” (Student 37)
**v. The Problem of a Dependence-Inducing Therapist**		“Users may overestimate the therapeutic benefits and underestimate the limitations of using such technologies, further deteriorating their mental health. Such a phenomenon can be classified as a therapeutic misconception where users may infer the chatbot’s purpose is to provide them with real therapeutic care.” (Student 21)
**vi. The Problem of an Anonymous but Impersonal Therapist**		“The reason why therapy sessions are always face to face is due to the fact that the body always betrays what the words don’t say; many physical cues will be missed if the person can select what to disclose to the AI without showing any weakness at all. That defeats the purpose of therapy.” (Student 41)

## Data Availability

The datasets used and/or analysed during the current study are available from the author upon reasonable request.

## Abbreviations

GenAI	Generative AI
AI	Artificial intelligence
AGI	Artificial general intelligence
ANI	Artificial narrow intelligence
