# Correction: Chan (2025). AI as the Therapist: Student Insights on the Challenges of Using Generative AI for School Mental Health Frameworks. *Behavioral Sciences*, *15*(3), 287

**DOI:** 10.3390/bs15030375

**Published:** 2025-03-17

**Authors:** Cecilia Ka Yuk Chan

**Affiliations:** Faculty of Education, The University of Hong Kong, Hong Kong SAR, China; ckchan09@hku.hk

In the original publication (Chan, 2025), “(Gaffney et al., 2020)” was not cited. The citation has now been inserted in “4.5. The Role and Limitations of Direct User Feedback in Evaluating AI Therapy”, “Paragraph 3” and should read as follows:

“Additionally, from a perceptual control theory perspective, therapeutic effectiveness is not solely about initial engagement but rather about prolonged, iterative exploration of issues that lead to deeper self-understanding and behavioural change. AI chatbots, such as MYLO, have attempted to integrate this model by encouraging users to re-engage with their issues across multiple interactions (Gaffney et al., 2020). However, without sustained emotional attunement, AI-driven therapy may struggle to replicate the long-term restructuring process that occurs in human-led interventions.”

“Gaffney, H., Mansell, W., & Tai, S. (2020). Agents of change: Understanding the therapeutic processes associated with the helpfulness of therapy for mental health problems with relational agent MYLO. *Digital Health*, *6*, 2055207620911580. https://doi.org/10.1177/2055207620911580.”

With this correction, the order of some references has been adjusted accordingly.

The authors state that the scientific conclusions are unaffected. This correction was approved by the Academic Editor. The original publication has also been updated.
